# Vitamin B_12_ Uptake by the Gut Commensal Bacteria *Bacteroides thetaiotaomicron* Limits the Production of Shiga Toxin by Enterohemorrhagic *Escherichia coli*

**DOI:** 10.3390/toxins8010014

**Published:** 2016-01-05

**Authors:** Charlotte Cordonnier, Guillaume Le Bihan, Jean-Guillaume Emond-Rheault, Annie Garrivier, Josée Harel, Grégory Jubelin

**Affiliations:** 1 INRA, UR454 Microbiologie, F-63122 Saint-Genès-Champanelle, France; charlotte.cordonnier@udamail.fr (C.C.); annie.garrivier@clermont.inra.fr (A.G.); 2 Groupe de Recherche sur les Maladies Infectieuses du Porc (GREMIP), Centre de Recherche en Infectiologie Porcine et Avicole (CRIPA), Université de Montréal, Faculté de Médecine Vétérinaire, C.P. 5000, Saint-Hyacinthe, QC J2S 7C6, Canada; guillaume.le.bihan@umontreal.ca (G.L.B.); jeang.emond6@gmail.com (J.-G.E.-R.); josee.harel@umontreal.ca (J.H.)

**Keywords:** enterohemorrhagic *Escherichia coli*, Shiga toxin, *Bacteroides thetaiotaomicron*, vitamin B_12_

## Abstract

Enterohemorrhagic *Escherichia coli* (EHEC) are foodborne pathogens responsible for the development of bloody diarrhea and renal failure in humans. Many environmental factors have been shown to regulate the production of Shiga toxin 2 (Stx2), the main virulence factor of EHEC. Among them, soluble factors produced by human gut microbiota and in particular, by the predominant species *Bacteroides thetaiotaomicron* (*B. thetaiotaomicron*), inhibit Stx2 gene expression. In this study, we investigated the molecular mechanisms underlying the *B. thetaiotaomicron*-dependent inhibition of Stx2 production by EHEC. We determined that Stx2-regulating molecules are resistant to heat treatment but do not correspond to propionate and acetate, two short-chain fatty acids produced by *B. thetaiotaomicron*. Moreover, screening of a *B. thetaiotaomicron* mutant library identified seven mutants that do not inhibit Stx2 synthesis by EHEC. One mutant has impaired production of BtuB, an outer membrane receptor for vitamin B_12_. Together with restoration of Stx2 level after vitamin B_12_ supplementation, these data highlight vitamin B_12_ as a molecule produced by gut microbiota that modulates production of a key virulence factor of EHEC and consequently may affect the outcome of an infection.

## 1. Introduction

The human gut is colonized by microbiota that is a dense population of microorganisms, primarily bacteria belonging to two major phyla: *Bacteroidetes* and *Firmicutes*. Gut microbiota provides many benefits for humans, including the digestion of food and production of end-products available for the host; production of key vitamins and hormones; and development of the immune system [1]. Gut microbiota efficiently limits infection by intestinal pathogenic bacteria, a phenomenon termed colonization resistance [2]. This concept is supported by numerous studies showing disruption of the normal microbiota. Antibiotic treatment, for example, leads to animals with a higher susceptibility to enteric pathogens [3,4,5]. To date, the molecular basis for colonization resistance remains elusive and relies on four possible mechanisms [6]. First, the microbiota can directly inhibit pathogen growth through the production of inhibitory metabolites and bacteriocins. Second, its ability to consume a very high variety of nutrients gives rise to gut resource depletion that is incompatible with the development of incoming bacteria. Third, mucosal surface–microbiota interactions stimulate the immune system. This may induce the release of antimicrobial peptides or secretory IgA providing host defense features against enteric pathogens. Fourth, activities of the gut microbiota have been shown to decrease the virulence properties of pathogens through the modification of expression levels for virulence genes.

EHEC, particularly those belonging to the O157:H7 serotype, are intestinal pathogens responsible for foodborne infections in humans. EHEC infection leads to the development of various disorders ranging from watery or bloody diarrhea to life-threatening diseases such as hemolytic and uremic syndrome or thrombotic thrombocytopenic purpura. Two cardinal virulence factors of EHEC are the type 3 secretion system (T3SS) and Stx. T3SS, encoded by the Locus of Enterocyte Effacement (LEE), is involved in the formation of attaching and effacing (A/E) lesions on the colonic epithelium [7] through the injection of specific effectors into epithelial cells. Interactions of bacterial effectors with eukaryotic signal transduction pathways lead to host cytoskeleton reorganization that is characterized by two key markers of A/E lesions: an effacement of microvilli and formation of pedestals beneath adherent EHEC [7]. Stx, the other critical virulence factor of EHEC, is encoded by genes located on lysogenic phages. Stx is an AB_5_ toxin that binds specifically to a receptor at the surface of intestinal and glomerular endothelial cells. Stx blocks translation in intoxicated cells resulting in cell death by apoptosis and renal dysfunction [8].

During the course of infection, EHEC encounter the intestinal environment and its microbiota. Several studies have clearly illustrated that EHEC create an adaptive response at the transcriptional level to successfully colonize the host, including activation of pathways such as neoglucogenesis, ethanolamine and mucus-derived sugar catabolism [9,10,11,12,13,14]. In response to intestinal signals, EHEC also modulate expression and/or activity of its virulence factors. We recently determined that LEE gene expression is significantly reduced by microbial activities in human gut microbiota-associated rats [13]. The two host hormones, epinephrine and norepinephrine, were also identified as signals sensed by EHEC to adjust the expression level of LEE genes [15]. Production of Stx and its translocation across the intestinal barrier are also subject to regulation by digestive molecules [16,17]. Transcriptional inhibition of Stx2-encoding genes is mediated by unknown molecule(s) produced by commensal bacteria of the gut including a predominant species of the phylum *Bacteroidetes*, *Bacteroides thetaiotaomicron* (*B. thetaiotaomicron*) [16].

The goal of our study was to identify molecules involved in the repressive effect of *B. thetaiotaomicron* on Stx2 production. Screening of a *B. thetaiotaomicron* mutant library uncovered several mutants that are unable to repress the synthesis of Stx2. Interestingly, one of these mutants has the transposon inserted into a gene encoding a specific transporter of vitamin B_12_ (also known as cobalamin). Together with restoration of Stx synthesis by cobalamin supplementation, our results highlight vitamin B_12_ as an additional digestive molecule that modulates production of virulence factors in EHEC and consequently may affect the outcome of an infection.

## 2. Results

### 2.1. Stx2-Repressing Molecules Are Heat-Resistant but Do Not Correspond to the Short-Chain Fatty Acids Produced by B. thetaiotaomicron

**Figure 1:**
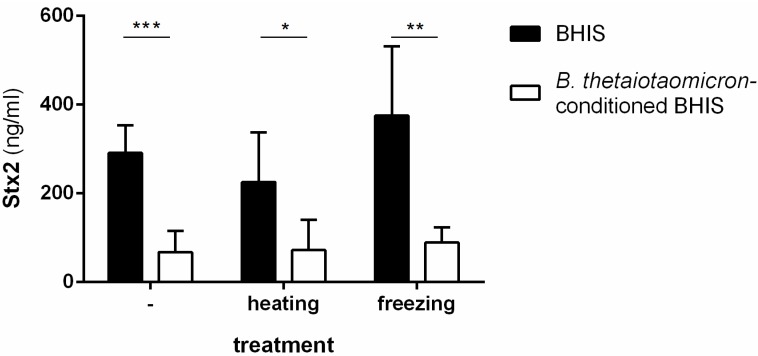
Biochemical characterization of Stx2 inhibitory molecules produced by *B. thetaiotaomicron*. Stx2 concentrations were measured after 6 h of growth of EDL933 in BHIS or in *B. thetaiotaomicron*-conditioned BHIS treated 15 min at 95 °C (heating) or 24 h at −80 °C (freezing) or left untreated as indicated. Student’s *t*-test: * *p* < 0.05, ** *p* < 0.01, *** *p* < 0.001.

To characterize biochemical properties of the Stx2 inhibitory molecule(s) produced by *B. thetaiotaomicron*, we subjected supernatants from culture of the commensal species in supplemented brain-heart infusion medium (BHIS) to either a 95 °C heat treatment or to storage at −80 °C before use as the growth medium for the EHEC O157:H7 strain EDL933. Production of Stx2 remained inhibited after growth of EHEC in all *B. thetaiotaomicron*-conditioned media (Figure 1), indicating that the inhibitory compounds are unaltered in these conditions. 

**Figure 2:**
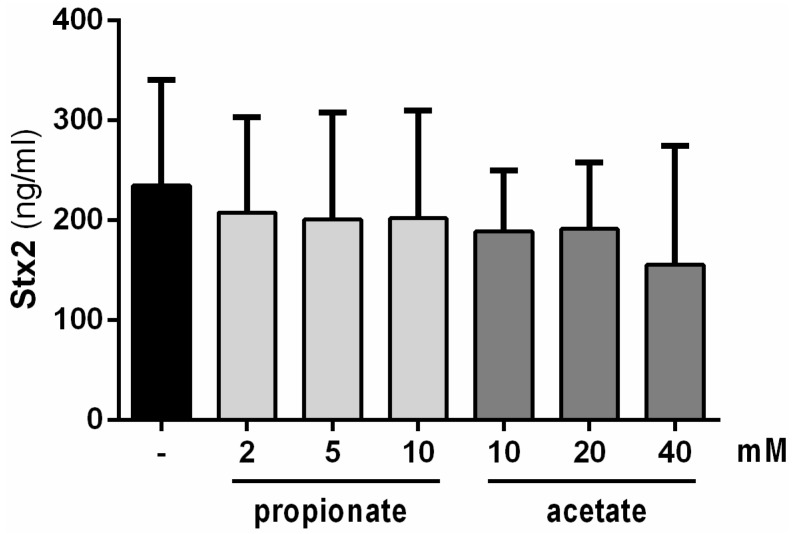
Impact of propionate and acetate on Stx2 production levels. Stx2 concentrations were measured after 6 h of growth of EDL933 in BHIS supplemented or not with indicated concentrations of SCFAs. No significant differences were observed. ANOVA: *p* > 0.05.

Our previous study demonstrated that the Stx2 inhibitory molecules have a molecular size less than 3 kDa [16]. Short-chain fatty acids (SCFAs), the end-products of fermentation of dietary fibers by gut microbiota, are small heat-resistant molecules and one of them, butyrate, regulates LEE gene expression in EHEC [18]. We investigated the potential effect of SCFAs produced by *B. thetaiotaomicron* (specifically acetate and propionate) on Stx2 production level. The range of tested concentrations for both SCFAs were selected based on experimental measurements after *in vitro* growth of *B. thetaiotaomicron* or from the digestive tract of *B. thetaiotaomicron* mono-associated rodents [19,20]. Figure 2 shows no differences in Stx2 production levels in the absence or presence of propionate or acetate under any concentration used. This result indicates that propionate and acetate have no impact on toxin synthesis under these conditions.

### 2.2. Screening of A B. thetaiotaomicron Mutant Library Identifies Seven Mutants that Lost Their Ability to Inhibit Stx2 Production by EHEC

**Figure 3:**
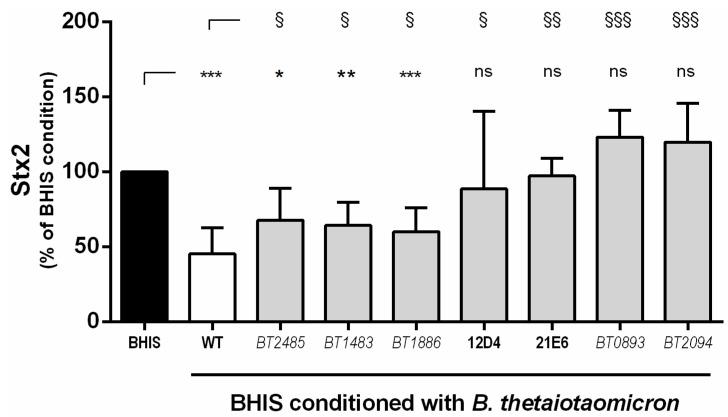
Seven *B. thetaiotaomicron* mutants have lost their ability to repress Stx2 synthesis. Stx2 concentrations were measured after 6 h of growth of EDL933 in BHIS or in BHIS conditioned with wild-type *B. thetaiotaomicron* or seven transposon-inserted mutants. ANOVA: ns *p* > 0.05, * *p* < 0.05, ** *p* < 0.01, *** *p* < 0.001 for BHIS versus conditioned media; ^§^ *p* < 0.05, ^§§^ *p* < 0.01, ^§§§^ *p* < 0.001 for wild-type-conditioned versus mutant-conditioned media.

To find genes involved in the production of Stx2 inhibitory molecules, a library of 4000 transposon mutants was generated using the *B. thetaiotaomicron* strain VPI-5482 (see Experimental Section for details). Mutants were cultivated individually to prepare conditioned media and Stx2 production levels were quantified after growth of EHEC strain EDL933. Media conditioned by eleven mutants led to higher level of Stx2 synthesis than in medium conditioned by wild-type *B. thetaiotaomicron*. To verify that these eleven clones were *B. thetaiotaomicron* isolates with integrated Tn4351 in their genome, we performed PCR controls using primers specific to a *B. thetaiotaomicron* gene or primers targeting *ermF* gene from transposon Tn4351. Of the eleven mutants, four were discarded after the PCR controls. We determined that the remaining seven *B. thetaiotaomicron* mutants lost their capacity to limit the production of Stx2 by EHEC (Figure 3) either partially or totally. Among them, three mutants demonstrated a repressive effect that was less strong than one with wild-type *B. thetaiotaomicron*. For these mutants, Tn4351 was inserted into gene BT_2485 encoding the major outer membrane porin OmpA, gene BT_1483 involved in the synthesis of sensor protein PhoR from the two-component system PhoBR or gene BT_1886, encoding a protein of unknown function with a domain similar to 2-nitropropane dioxygenase. Another four *B. thetaiotaomicron* mutants fully lost their capacity to inhibit the synthesis of Stx2 by EDL933. Indeed, amounts of toxin detected in BHIS conditioned with these mutants were not significantly different from those obtained following the growth of EHEC in unconditioned BHIS (Figure 3). Despite our efforts, transposon insertion sites were not identified for clones 12D4 and 21E6. For two other clones, genes BT_0893 or BT_2094, were inactivated by the integration of Tn4351. BT_0893 corresponds to gene *trmD* that directs the synthesis of a tRNA-methyltransferase and BT_2094 encodes a functional outer membrane transporter of vitamin B_12_ [21].

### 2.3. Vitamin B_12_ Activates the Production of Stx2 by EHEC

**Figure 4:**
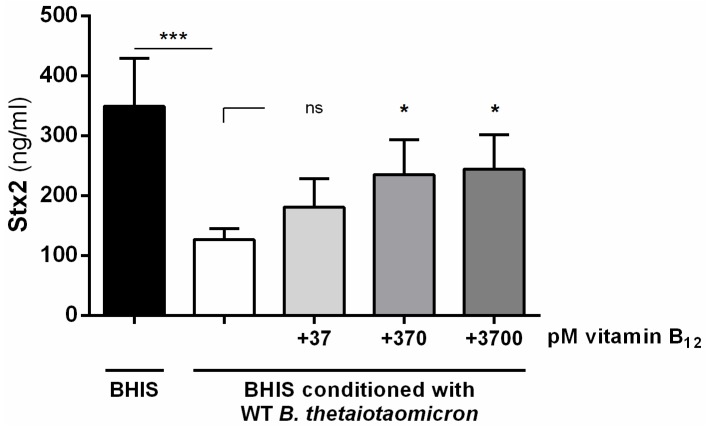
Vitamin B_12_ induces the production of Stx2. Stx2 concentrations were measured after 6 h of growth of EDL933 in BHIS or in wild-type *B. thetaiotaomicron*-conditioned BHIS supplemented or not with indicated concentrations of vitamin B_12_. ANOVA: ns *p* > 0.05, * *p* < 0.05, *** *p* < 0.001.

By inactivating gene BT_2094 in *B. thetaiotaomicron*, the commensal bacterium is unable to repress the production of Stx2 by EHEC. We focused our attention on this mutant because: (i) gene BT_2094 has been functionally described (renamed *btuB3*, a term used hereafter) as a gene encoding one of the three transporters of vitamin B_12_ produced by *B. thetaiotaomicron* [21]; (ii) vitamin B_12_ is a key vitamin for the host and is exclusively produced by prokaryotes [22]; and (iii) it has been well described that vitamin B_12_ affects gene expression in bacteria via riboswitches [23]. To explore the potential role of vitamin B_12_ in the *B. thetaiotaomicron*-dependent inhibition of Stx2, we first quantified vitamin B_12_ in BHIS before and after the growth of wild-type or *btuB3* strains of *B. thetaiotaomicron*. As expected, the wild-type strain consumed vitamin B_12_ during its growth in BHIS (551 ± 73 pM *vs.* 92 ± 8 pM in BHIS before and after the growth, respectively; *p* < 0.0001 Student’s *t*-test). While the *btuB3* mutant consumed a large amount of vitamin B_12_, concentration of the remaining vitamin B_12_ into the medium after growth was slightly but significantly higher with the *btuB3* mutant than with the wild-type strain (105 ± 4 pM *vs.* 92 ± 8 pM in BHIS, respectively; *p* < 0.05 Student’s *t*-test). This indicates that inactivation of *btuB3* partially affects the capacity of *B. thetaiotaomicron* to internalize vitamin B_12_. Next, we performed Stx2 quantification after the growth of EDL933 in BHIS conditioned with wild-type *B. thetaiotaomicron* and supplemented with different concentrations of cobalamin. Figure 4 shows that the addition of exogenous vitamin B_12_ led to a significant dose-dependent increase in Stx2 production. This indicates that vitamin B_12_ is an inducer of Stx2 synthesis and suggests that its consumption by *B. thetaiotaomicron* affects the level of toxins produced by EHEC. It is noteworthy that restoration remained partial even with the highest concentration of vitamin B_12_ since the amount of produced Stx2 was not as high as in unconditioned medium. Because vitamin B_12_ can modulate gene expression through binding to riboswitches, we searched the genome of EDL933 for a B_12_ riboswitch using the RFAM search tool. The only detected B_12_ riboswitch was the well characterized riboswitch located in the 5’-UTR of the *btuB* gene. This suggests that the influence of vitamin B_12_ on Stx2 production by EHEC may not be mediated by a B_12_ riboswitch-dependent regulation mechanism.

## 3. Discussion

In this study, we investigated the repressive effect of *B. thetaiotaomicron* on the ability of EHEC to produce Stx2. Screening of a *B. thetaiotaomicron* mutant library revealed that a mutant affected in the production of the vitamin B_12_ transporter BtuB3 lost its ability to inhibit the synthesis of Stx2. Properties of vitamin B_12_ are compatible with previous data showing that Stx2-affecting molecules recovered from the supernatant of *B. thetaiotaomicron* culture were resistant to heat treatment and were less than 3 kDa in size (this study and [16]). In addition, supplementation of wild-type *B. thetaiotaomicron*-conditioned medium with cobalamin partially restores the production of Stx by EHEC. This indicates that vitamin B_12_ stimulates EHEC to produce Stx2 and that growth of *B. thetaiotaomicron* inhibits toxin synthesis probably through medium depletion of vitamin B_12_. Quantification of vitamin B_12_ in BHIS after the growth of *B. thetaiotaomicron* clearly demonstrated that the commensal bacterium internalizes the vitamin, which is required for the function of many key enzymes including the methionine synthase [24]. The *btuB3* mutant was also able to uptake cobalamin but with slightly reduced efficiency compared to the wild-type strain. This difference is probably too small to explain by itself the inability of the *btuB3* mutant to repress Stx2 synthesis by EHEC. Recently, Degnan and coworkers showed that *B. thetaiotaomicron* uses three functional vitamin B_12_ transporters (named BtuB1–3) that exhibit specific preferences to distinct B_12_ analogs [21]. BtuB3 has a greater affinity to B_12_ analogs containing 2-methyladenine, 5-methoxybenzimidazole, 5-methylbenzimidazole, or 5,6-dimethylbenzimidazole as the lower ligand linked to cobinamide [21]. Our vitamin B_12_ quantification method does not provide distinction between the different forms of cobalamin. We speculate that the *btuB3* mutant is unable to uptake some particular B_12_ analog(s) that might specifically affect Stx2 synthesis by EDL933.

Few studies have directly investigated the role of vitamin B_12_ in the virulence of pathogens. Whereas inactivation of the vitamin B_12_ uptake or synthesis pathway does not affect the virulence of *Salmonella* *enterica* (*S. enterica*) serovar Typhimurium in mice [25], a defective mutant in vitamin B_12_ production of *S. enterica* serovar Gallinarum, a fowl pathogen, is avirulent in chickens [26]. Vitamin B_12_ also affects the virulence of *Listeria monocytogenes* in mice through the binding to a riboswitch element controlling the expression of a small non-coding RNA, Rli55 [27]. Rli55 sequesters and inhibits the action of EutV, a transcriptional activator of the *eut* operon required for the use of ethanolamine, an abundant substrate in the gut of mammalians. If the *eut* operon exists in EHEC, it differs from the one in *Listeria*, especially for the regulatory network controlling its expression. In addition, no B_12_ riboswitch element was found in the genome of EHEC EDL933 except the well described riboswitch regulating the expression of the *btuB* gene. Ethanolamine catabolic pathways are widely conserved in gut pathogenic bacteria [28] and have been involved in the virulence of several [27,29,30,31,32]. Interestingly, vitamin B_12_ is essential for ethanolamine degradation since it is a co-factor of the enzyme ethanolamine ammonia-lyase [33]. Moreover, the main activator of *eut* genes in *Escherichia* and *Salmonella* species, EutR, also regulates the expression of other genes related to virulence. Indeed, Luzader and coworkers demonstrated that EutR activates LEE gene expression in EHEC O157:H7 in an ethanolamine-dependent manner [34]. Although there is no direct evidence, vitamin B_12_ is probably a co-factor of EutR since vitamin B_12_ with ethanolamine is required by EutR for full activation of *eut* and LEE operons [34,35,36]. Production of Stx2 has been determined to be also affected by the presence of ethanolamine in the environment [29]. This effect occurs, at least partially, in a EutR-dependent way. Such might explain why the consumption of vitamin B_12_ by *B. thetaiotaomicron* leads to a decrease in Stx2 production levels by EHEC.

With vitamin B_12_ supplementation even in high concentration, full restoration of Stx2 synthesis is not achieved. These data suggest that Stx2 modulation by *B. thetaiotaomicron* is certainly multifactorial. We have determined that acetate and propionate, the SCFAs produced by *B. thetaiotaomicron*, have no impact on Stx2 production. However, acetate has been proposed to affect the translocation of Stx2 across intestinal mucosa [17], suggesting that *B. thetaiotaomicron* may provide protection from an EHEC infection at different levels. In contrast, Crane and coworkers have shown that EHEC infection triggers the production of xantine oxidase by intestinal epithelial cells [37]. Xantine oxidase activity increases the ability of Stx2 to translocate across intestinal monolayers and leads to the production of hydrogen peroxide that negatively impacts commensal bacteria, including *B. thetaiotaomicron*. Taken together, these data illustrate that EHEC and *B. thetaiotaomicron* are mutually antagonistic and that the outcome of interactions between the two species may contribute important consequences to disease progression.

The *phoR* mutant (gene BT_1483), is one of the *B. thetaiotaomicron* mutants that is unable to repress the production of Stx2. PhoR is the sensor protein of the PhoBR two-component system involved in the regulation of many genes in response to the concentration of extracellular inorganic phosphate [38]. Disruption of the PhoBR system disallows the bacterium to properly regulate phosphate internalization. Interestingly, a recent report has shown that production of Stx2 by EHEC is modulated by the concentration of extracellular inorganic phosphate [39]. This suggests that both an effective regulation of phosphate uptake and assimilation by *B. thetaiotaomicron* might also have important roles in protection against Stx2 production by EHEC.

In this study, we have investigated how *B. thetaiotaomicron* affects the production of Stx2 by EHEC. We have demonstrated that the two SCFAs, acetate and propionate, produced by this gut commensal bacterial species, have no effect on toxin synthesis. In contrast, a mutant of *B. thetaiotaomicron* with impaired production of a specific transporter of vitamin B_12_ is no longer able to inhibit the production of Stx2. This work suggests that concentration of vitamin B_12_ in the gut and by extension, activities of commensal bacterial species producing and/or consuming vitamin B_12_, may modulate the production of the main virulence factor of EHEC. Our research provides an additional element to the growing list of environmental cues EHEC can sense in the gut during the infectious process in order to successfully manage its virulence program.

## 4. Experimental Section

### 4.1. Bacterial Strains, Media and Growth Conditions

The human gut commensal bacteria *B. thetaiotaomicron* VPI-5482 was grown in BHIS medium [40] under anaerobic conditions at 37 °C. The *E. coli* strain WM3064 used for mating experiments was grown in Lysogeny broth (LB) supplemented with 100 µM of 2,6-diamino-pimelic acid. Wild-type EHEC O157:H7 strain EDL933 was grown in LB medium, in BHIS or in *B. thetaiotaomicron*-conditioned BHIS as described previously [16]. Briefly, *B. thetaiotaomicron*-conditioned BHIS was prepared after a 24-h growth of the commensal strain in BHIS. After culture centrifugation, the supernatant, filtered through 0.2 µm filters was supplemented with concentrated LB to reach a final concentration of 0.5 *X*. pH was adjusted to 7.0 and the medium was filtered with a 0.2-µm filter. When required, acetate or propionate (pH 7.0) were added to BHIS medium with indicated concentrations; antibiotics were used at the following concentrations: 10 µg·mL^−1^ erythromycin; 50 or 200 µg·mL^−1^ gentamycin; 25 µg·mL^−1^ chloramphenicol; and 10 µg·mL^−1^ tetracycline.

### 4.2. Construction and Screening of the B. thetaiotaomicron Mutant Library

The transposon Tn4351 was used to construct an insertion mutant library in *B. thetaiotaomicron* strain VPI-5482 as described previously [41]. The suicide vector pEP4351 carrying the transposon Tn4351 was transferred to *B. thetaiotaomicron* by mating experiments using WM3064 as the donor strain. After independent growth, donor and recipient strains were mixed at ratio 1:1 or 1:5, respectively, and spotted on a nitrocellulose filter placed on a BHIS + 2,6-diamino-pimelic acid agar plate. After an overnight incubation at 37 °C under aerobic condition, bacteria were recovered from filters and washed twice with BHIS. After dilution, bacteria were plated on BHIS + erythromycin and incubated under anaerobic conditions for 48 h at 37 °C in order to select *B. thetaiotaomicron* transconjugants. 

To identify *B. thetaiotaomicron* mutants that no longer repress the production of Stx2 by EHEC, the mutant library was screened as follows: first, each mutant was grown in 1 ml of BHIS in Deepwell 96 microplates for 48 h. The plates were centrifuged and supernatants were transferred to new Deepwell microplates. After supplementation with LB (0.5 *X* final) and a determined volume of NaOH to yield a neutral pH, the *B. thetaiotaomicron*-conditioned media were inoculated with EDL933 strain and incubated for 6 h with orbital agitation. Controls were included in each plate; EDL933 was cultivated in BHIS and BHIS conditioned with wild-type *B. thetaiotaomicron*. The plates were centrifuged and supernatants were collected to perform Stx2 quantification. Selected mutants were checked by PCR using *B. thetaiotaomicron* specific primers BT-F (5’-AACAGGTGGAAGCTGCGGA-3’) and BT-R (5’-AGCCTCCAACCGCATCAA-3’) ([42]) or primers 341 (5’-TTGGAAATTTTCTGGGAGG-3’) and Tn4351-Rev (5’-TAACCATTGACTTGGAGAC-3’), targeting *ermF* gene from Tn4351. Identification of transposon insertion regions was performed using arbitrarily primed PCR as described previously [43].

### 4.3. Biochemical Methods

Amounts of Stx2 in culture supernatants were quantified using an enzyme-linked immunoassay as previously described with slight modifications [44]. Sandwich ELISAs were performed in 96-well plates using a monoclonal antibody against Stx2 (Stx2-BB12, Toxin Technology, Sarasota, FL, USA) diluted 1:1600 to coat the plates. Two-fold serial dilutions of samples were incubated in coated plates. When indicated, samples were beforehand treated 15 min at 95 °C or 24 h at −80 °C. Rabbit polyclonal antiserum against Stx2 diluted 1:1000 was used to detect the toxin. A standard curve was obtained with several dilutions of purified Stx2 (Toxin Technology, Sarasota, FL, USA). Detection was performed with a 1: 5 000 dilution of horseradish peroxidase-goat IgG anti-rabbit (ThermoFisher Pierce, Waltham, MA, USA) and *o*-phenylenediamine stable peroxidase substrate (OPD, Sigma-Aldrich, St. Louis, MO, USA).

The concentration of vitamin B_12_ in BHIS was analyzed using the Dimension VISTA immunoassay system and the analytical kit supplied by the manufacturer (Siemens Healthcare, Erlangen, Germany).

### 4.4. Statistical Analysis

All data represent the mean ± the standard deviation of at least three independent experiments. Student’s *t*-test or ANOVA with Fisher’s LSD test were used to determine significant differences between two or several test groups, respectively as shown in the figures.
